# The development and effectiveness of web-based psychological capital intervention on the mental well-being of tourism workers during the COVID-19 pandemic

**DOI:** 10.1186/s40359-023-01189-0

**Published:** 2023-04-28

**Authors:** Thanayot Sumalrot, Charin Suwanwong, Saran Pimthong, Amaraporn Surakarn, Araya Chiangkhong, Anon Khunakorncharatphong

**Affiliations:** 1 Faculty of Medicine Siriraj Hospital, Mahidol University, Bangkok, Thailand; 2 Behavioral Science Research Institute, Srinakharinwirot University, Bangkok, Thailand; 3 Graduate School, Srinakharinwirot University, Bangkok, Thailand; 4 Kuakarun Faculty of Nursing, Navamindradhiraj University, Bangkok, Thailand; 5 International Health Policy Program (IHPP), Ministry of Public Health, Nonthaburi, Thailand

**Keywords:** COVID-19, Mental health, Mental well-being, PCI, Psychological capital, Tourism business, Web-based intervention

## Abstract

The current COVID-19 situation has forced many tourism businesses to close. Tourism workers have suffered chronic stress, discouragement, despair, and failure to find solutions for their businesses, resulting in deteriorating mental health. A psychological capital intervention (PCI) is therefore deemed necessary as it promotes the mental well-being of tourism workers. This article reports the development of a web-based PCI for the mental well-being of tourism workers and an investigation of its effectiveness using a mixed-methods intervention design. A qualitative approach was used to develop the intervention by conducting interview techniques with 20 tourism entrepreneurs. A content analysis was carried out. The intervention was tested through an experimental research design. The sample comprised 600 tourism workers who undertook a web-based PCI. Data on their mental well-being were collected before the intervention and 1 month afterward and analyzed using ANCOVA. The research revealed that 4 themes are needed for the intervention: having a goal, tolerance of difficulties, inspiration from a role model, and the appropriate way of thinking. An effectiveness examination showed that the web-based PCI improves mental well-being scores significantly. In conclusion, this web-based PCI, which focuses on developing strengths, effectively improves the mental well-being of tourism workers facing difficulties during the COVID-19 pandemic.

## Introduction

Thailand has been facing the pandemic of coronavirus disease 2019 (COVID-19), an acute respiratory disease highly contagious among humans. The outbreak was sudden and critical. The situation has gravely affected businesses in several ways and is very challenging to control. Business operations have been forced to halt, especially in the tourism sector [1]. Due to the viral pandemic, the number of foreign tourists visiting Thailand in the first quarter of 2020 decreased by 38% relative to the corresponding period in 2019. As a result, the revenue from foreign tourists amounted to 332 billion baht, representing a 40% decline from the same period in 2019. In addition, domestic travel saw a 31% drop compared with the same period of the preceding year, leading to a 32% decrease in spending by domestic tourists. Such statistics signal a continuous and increasingly severe economic contraction with the prolonging of the pandemic. The Thai government has imposed a wide range of control measures. They include mandatory closure of high-risk businesses and tourist attractions, a ban on unnecessary cross-provincial travel, and a requirement for businesses to implement the government’s work-from-home policy [2]. Likewise, Marome and Shaw [3] commented that despite effective control of the spread of COVID-19, the overall crisis was poorly managed, resulting in adverse economic and social consequences. The services and tourism industries are most affected as they are financially insecure, and the initial financial support from the government failed. Consequently, achieving economic recovery in the services and tourism sectors is particularly challenging. Pongsakornrungsilp et al. [4] added that many tourism businesses had to cease operations. Tourism workers have been extensively affected because they generally did not receive financial compensation after being forced to leave their jobs. On the other hand, those who could continue working in the tourism industry have suffered from chronic stress, discouragement, and despair, resulting in deteriorating mental health [5, 6].

Mental well-being development programs have been launched for tourism entrepreneurs to address mental health problems and improve their mental health. In well-being studies, 2 pillars of well-being are widely recognized. One is “Hedonic,” which concerns experiences such as pleasure and satisfaction with life. The other is “Eudaimonic.” This pillar focuses on positive functioning through positive relationships and self-realization, which can lead to self-development, self-acceptance, and autonomy [7]. Mental well-being involves integrating both concepts to achieve a flourishing and good life [8]. The COVID-19 epidemic has significantly affected the health of tourism workers [9, 10]. Fear of economic crises and perceptions of job insecurity are the major causes of poor mental health among tourism workers [11]. It is therefore crucial to relieve the mental health problems that arise among tourism workers after facing the COVID-19 situation. The development of mental well-being is a way to help people maintain a positive psychological state amidst the current pandemic and to cope with any other crises that may arise.

One of the core factors contributing to the development of mental well-being or better mental health is positive psychological capital (PsyCap) [12–19]. The concept of PsyCap, introduced by Luthans et al. [20], refers to a person’s positive psychological state of development. It consists of 4 key elements:

- self-efficacy: the confidence to succeed in challenging tasks,
- optimism: making attributions about success in the present and the future,
- hope: perseverance to achieve a goal and find a pathway to success, and.
- resilience: the ability to bounce back and become stronger by overcoming difficult situations.

Luthans and Youssef-Morgan [21] described PsyCap as a state-like resource and an openness to development and change. Therefore, PsyCap is a positive attribute. It signifies an individual’s strength, and it can lead to the development of individual work and the promotion of well-being. Improving PsyCap requires improvement to all 4 of its key elements. For example, hope can be developed through effective goal setting, contingency planning, practicing the setting of challenging goals, and becoming aware of how to achieve goals. Self-efficacy can be improved through mastery experience, observational learning, social persuasion, and positive feedback. Resilience training can be undertaken through a combination of asset, risk, and process-focused approaches that emphasize the building and effective deployment of assets to alleviate risk factors. Last, optimism can be developed through positive self-talk and modification of past, present, and future thinking patterns to be more suitable. The improvements in each element are combined to develop PsyCap training. A study was conducted on PsyCap training for working-age adults [22]. However, there have been no studies on improving the mental well-being of tourism workers through a PsyCap intervention (PCI). Therefore, an intervention focusing on PsyCap training may improve the mental well-being of people working in the tourism industry.

However, the stagnant tourism industry and the COVID-19 pandemic make implementing a PCI in the field or with actual people arduous. Thanks to technological advancements and internet access, tourism workers are increasingly using the internet in their business operations. Therefore, a web-based PCI seems appropriate. Moreover, such an intervention is easily accessible and can be undertaken by many people. Previous studies have also confirmed that web-based PCIs are effective for personal development [23–25].

For these reasons, the authors considered that PsyCap might have the potential to play a critical role in enhancing the mental well-being of tourism workers. Development through the PsyCap concept will help to build and reinforce personal strengths, underpinning the mental discipline needed for effective and efficient business management. Gaining better mental health is metaphorically like wearing armor that protects tourism workers in new crises and supports the excellent physical and mental performance needed for business success. Therefore, this research aimed to develop a web-based PCI and investigate its effectiveness in improving the mental well-being of tourism workers.

## Methods

### Research design

This research drew upon a mixed-methods intervention design. The study consisted of 2 phases: development of the intervention using a qualitative approach and examination of the intervention’s effectiveness using an experimental design.

### Phase 1: development of web-based PCI

Using a qualitative approach, the authors developed content and activities based on the positive PsyCap concept that were suitable for the target group affected by the COVID-19 pandemic. A setting of this study is the main and famous province of Thailand’s tourism, which is an enterprise that operates a variety of businesses such as accommodation, leisure activities, night entertainment, and trade places that are affected by COVID-19 [2]. Twenty key informants were selected as the primary sources of data. The inclusion criteria were as follows: (1) being a tourism entrepreneur; (2) having at least 5 years of experience in tourism business operations; and (3) being affected by the COVID-19 pandemic, such as a lower income or a temporary shutdown. The participants who did not meet the inclusion criteria, had severe physical or mental problems, or were unable to give informed consent, were excluded. All included key informants were interviewed individually and in groups to explore the content and activities related to PsyCap that were suitable for tourism workers. Content analysis by frequency technique was used to identify and analyze patterns of words and themes. Methodological triangulation, both in individual and in group interviews, was used to ensure the creditability of these findings. The data was used to develop a web-based PCI. After the content of PCI intervention were developed, the intervention was validated by five experts and was tested its feasibility and appropriateness with a hundred of tourism workers. By doing this, advice and feedback can be obtained to improve the details of the PCI program such as activity contents, format, color tone, background music, etc.

### Phase 2: investigation of the effectiveness of web-based PCI

**Fig. 1:**
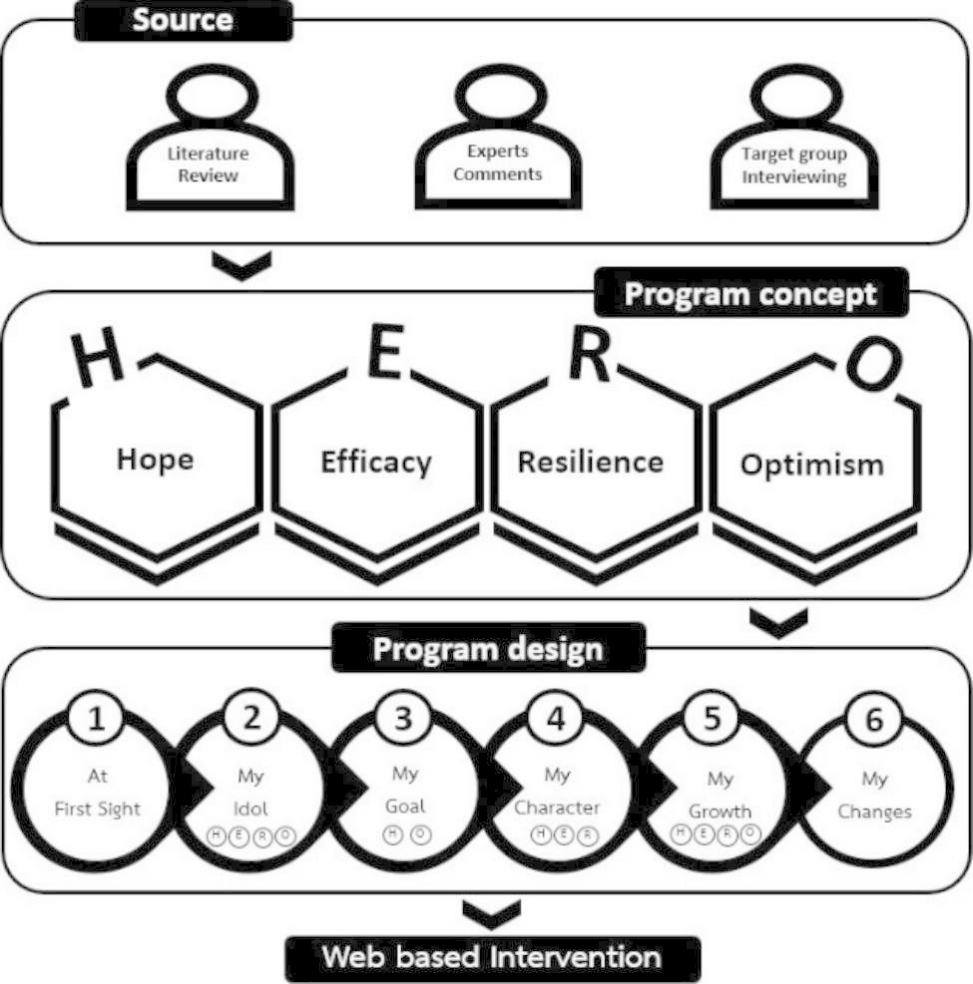
Development and design of the web-based PCI

This research used a 1-group, pretest-posttest design. The sample was drawn from tourism workers who were selected by using multistage random sampling (see Fig. 1). In the first stage, 1 province was selected from each of the 6 regions of Thailand. The selected provinces were popular tourist destinations: Chiang Mai (in the northern region), Nakhon Ratchasima (northeastern), Chon Buri (eastern), Prachuap Khiri Khan (western), Phuket (southern), and Bangkok (central). In the second stage, purposive sampling was applied to randomly select 100 tourism workers in each province (a total of 600 workers). The inclusion criteria were as follows: (1) being at least 18 years of age; (2) working in a tourism-related business (such as tour guiding, hotel services, transportation, conference services, food and beverage, entertainment, product sales, and massage and spa); and (3) having at least 1 year of work experience. Tourism workers were excluded in the study if they could not communicate in Thai or did not know how to use a mobile phone or a computer. A questionnaire was administered to collect the data needed to determine the effectiveness of the web-based PCI. All samples were informed of this availability of PCI program via the gate keeper of each selected province who distribute the URL of the intervention via online platform and monitor usage of the participants. All sample participated voluntarily by consent and answered the pre-test questionnaires for the first time, then, participate in the PCI web-based intervention and the post-test was given 1 month afterward, they received an e-mail to remind them to do a post-test to assess their mental well-being.

Th ethical approval for this study was obtained from the Human Research Ethics Committee, Strategic Wisdom and Research Institute of Srinakharinwirot University (certificate number SWUEC-209/2564E). All subjects signed a letter indicating their free and informed consent to participate in the study. The consent letters addressed the research objectives, voluntary participation, and the right to withdraw from the research at any time. They also stated that all information would be confidential and not disclosed. The individuals were also protected by research participants’ rights per the ethical criteria for conducting human research.

### Intervention

Based on our literature review, PsyCap consists of 4 components: “Hope,” “Self-Efficacy,” “Resilience,” and “Optimism,” abbreviated as “HERO.”

“Hope” entails setting objectives, creating a plan of action, and getting beyond barriers. “Self-efficacy” is a concept that includes mastery experience, vicarious learning, social persuasion, positive feedback, and physiological and psychological arousals. “Resilience” entails creating asset factors, overcoming risk factors, and engaging in an influencing process. Building efficacy or confidence and creating a positive outlook are also components of “Optimism.” People are urged to plan ahead for negative occurrences that could hinder them from accomplishing the objective and to consider how to lessen their effects. It’s thought that speaking well of oneself increases optimism.

We synthesized previous studies that used web-based PCI [23–25] to design our intervention. The activities included setting a specific, measurable, achievable, relevant, and time-bound (SMART) goal, dividing the goal into smaller goals, drilling to generate pathways to the goal, hearing the success story of others (role models), self-reflection, identifying strengths and weaknesses, sharing one’s goal with others, planning, and self-review.

### Instrument

The instrument used for collecting data was the Thai Mental Well-Being Scale developed by Pimthong et al. [26]. This scale was develop based on related concept of mental well-being and collected data from 2000 Thai working-aged people, and the results of the psychometric properties test were also good in terms of validity and reliability. The scale employs 10 items to evaluate mental well-being. This self-report instrument has 3 key elements: positive emotions and thinking (4 items); positive relationships (3 items); and positive functioning (3 items). A 6-point Likert scale is used, ranging from “strongly disagree” (1 point) to “strongly agree” (6 points). The scale has a Cronbach’s alpha reliability coefficient of 0.91. Individuals with high scores are more likely to have mental well-being than those with lower scores.

In addition, data were collected on how frequently intervention was used in tourism work. These data were collected after the experiment. In 1 item, participants were asked to assess the frequency of using the intervention in their tourism work by using a 5-point Likert scale ranging from “lowest” (1 point) to “highest” (5 points). Individuals with high scores were more likely to use the intervention in tourism work than those with lower scores.

### Data analysis

Data were analyzed using normal distribution, skewness, kurtosis, and the Kolmogorov–Smirnov Test. Normal distribution was demonstrated. The effectiveness of the web-based PCI was evaluated by using the pretest mental well-being score as a covariate and dividing the frequency of applying the intervention in tourism work into groups. The effectiveness of the intervention was analyzed with ANCOVA.

## Results

Phase 1 of the study revealed 4 themes for developing the PCI (Table 1). Subsequently, the draft of the PCI for tourism workers was examined for content validity by 5 experts in psychology and behavioral sciences, including stakeholders involved in tourism policymaking. They were asked to review the draft intervention and give feedback on the content and recommendations for improving the web-based intervention. Subsequently, we piloted in 100 samples to confirm its suitability.

**Table 1:** PCI themes and example quotations

Theme	Example of quotation
Having a goal	“I set a goal for myself to get some inspiration.” “I want my family to be here together.”
Tolerance to difficulties	“I have to learn to live with it. I have my child and my wife. They are still here with me. We need to encourage one another.” “I must fight and keep going.”
Inspiration from a role model	“Seeing other people gradually adapt to it, I am inspired to give it another go.” “Some people change jobs to become Grab riders and manage to carry on. I am hopeful and want to do the same.”
The appropriate way of thinking	“I don’t think COVID will last forever.” “I’m not alone in this crisis. I have many friends here.”

**Fig. 2:**
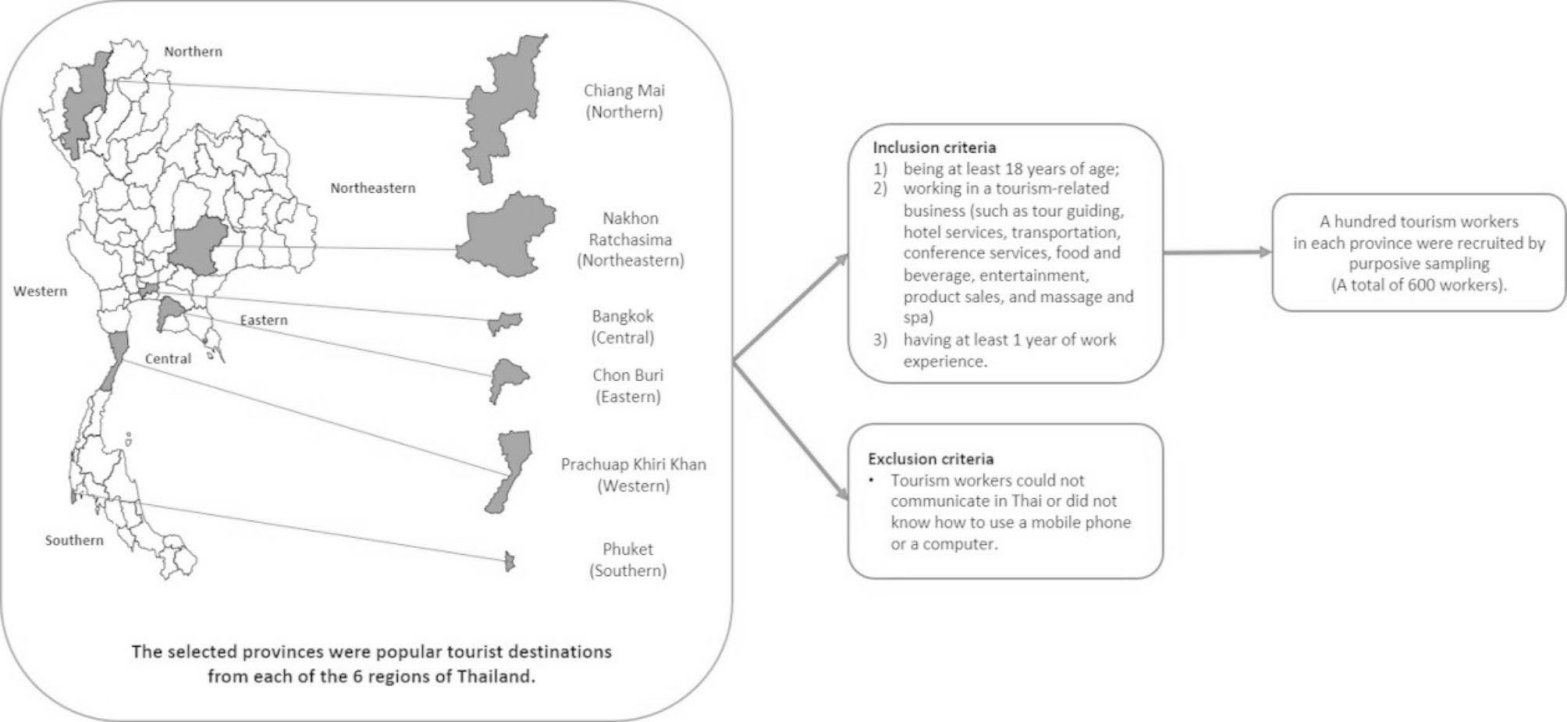
Overview of multistage random sampling

Next, the structure of the web-based PCI was derived (https://bsri2.swu.ac.th/myhero/). The web-based PCI consisted of 6 activities in a self-guided format. The activities were generally designed to facilitate participant self-reflection and self-review by answering questions presented as multiple choices, fill in blanks, and rating scales. The intervention also included video clips from role models and experts and easily comprehensible infographics. The first activity was entitled “At first sight.” It required individuals to self-report their mental well-being by answering short questions and to learn the importance of PsyCap according to the HERO components of PsyCap. The second activity was “My idol.” It involved creating inspiration for oneself by viewing video clips of role models and forwarding messages designed to encourage others who are experiencing a crisis. The third activity was “My goal.” It focused on designing and creating hope for oneself by setting a SMART goal. The fourth activity was “My character.” It explored an individual’s existing good attributes and the supporting factors that would help to solve their problems and handle the crisis. The fifth activity was “My growth.” It reviewed the SMART goal, summarized previous answers, and automatically generated scenes to stimulate positive feelings and provide support to overcome adversities. The final activity was entitled “My change.” It entailed evaluating psychological changes after the intervention to achieve sustainable improvement in an individual’s mental well-being. The activities are summarized in the Fig. 2.

For Phase 2 of the research, 600 participants were recruited. Their average age was 35.1 years (SD, 11.6; range, 18–73), and women predominated (64.0%). Most worked in food and beverage businesses (26.3%), with the selling of products ranking second (25.0%). Most participants were operational-level employees (44.8%), followed by entrepreneurs or owners (37.8%). Financial issues were mostly related to insufficient income for expenses (63.3%). More than half of the participants (63.0%) did not have adequate savings. Last, the large majority had debts (72.7%; Table 2).

**Table 2:** General and financial characteristics of the research participants (*N* = 600)

Sample characteristics	Number	Percent
Gender		
Male	216	36.0
Female	384	64.0
Business type		
Tour guiding	64	10.7
Accommodation	83	13.8
Transportation	43	7.2
Conference services	38	6.3
Food and beverage	158	26.3
Entertainment	36	6.0
Product sales	150	25.0
Massage and spa	28	4.7
Position		
Entrepreneurs or owner	227	37.8
Executive	104	13.7
Operational-level employee	269	44.8
Income		
Insufficient	380	63.3
Sufficient	220	36.7
Savings		
Have no savings	378	63.0
Have savings	222	37.0
Debt		
Do not have debts	164	27.3
Have debts	436	72.7

The overall mental well-being of the participants increased at a statistically significant level of 0.05. When analyzed by province, the data revealed similar results, with significantly increasing trends. Tourism workers in the province of Phuket showed the greatest improvement in mental well-being, followed in descending order of improvement by workers in Chon Buri, Chiang Mai, Nakhon Ratchasima, Bangkok, and Prachuap Khiri Khan. The effect size of the web-based PCI on the mental well-being change of the tourism workers was at the medium to high level (d = 0.44–1.10; Table 3).

**Table 3:** Pre- to post-intervention changes in the mental well-being of tourism workers (*N* = 600)

Target area	Pre-intervention		Postintervention		MD	*t*	*p*	Cohen’s d
	M	SD	M	SD				
Overall	41.16	8.11	46.44	8.20	5.29	16.37	< 0.001	0.65
- Bangkok	42.44	9.90	46.62	8.84	4.18	5.00	< 0.001	0.44
- Chon Buri	41.10	9.47	47.49	8.35	6.39	6.07	< 0.001	0.71
- Chiang Mai	41.58	7.75	47.77	7.50	6.19	8.90	< 0.001	0.80
- Nakhon Ratchasima	42.09	7.56	46.69	8.80	4.60	6.17	< 0.001	0.55
- Prachuap Khiri Khan	38.30	5.91	41.65	7.96	3.35	5.61	< 0.001	0.47
- Phuket	41.44	6.86	48.44	5.60	7.00	10.14	< 0.001	1.10

M, mean; SD, standard deviation; p, level of significance; d, effect size

An analysis was made of how the frequency of application to the participants’ daily life from the web-based PCI and how it affected the changes in the mental well-being scores of the tourism workers. For this purpose, analysis of covariance (ANCOVA) examined the pretest-posttest differences, controlling for the pretest mental well-being scores and grouping by frequency of application of the intervention (Table 4). Different frequencies of application of the intervention produced significantly different levels of change in the mental well-being of the tourism workers (F[4, 594] = 27.974; *P* = 0.000). Notably, the intervention application frequency was directly proportional to the mental well-being score. In other words, the tourism workers who applied the intervention more frequently had higher mental well-being scores than those who applied the program less frequently.

**Table 4:** Frequency of application of the skills from the web-based PCI for the mental well-being of tourism workers (*N* = 600)

Application frequency	n	M	SE	Change in mental well-being				
				Lowest	Low	Medium	High	Highest
Lowest	4	37.04	3.21	-	-	-	-	-
Low	10	39.08	2.03	2.05	-	-	-	-
Medium	158	43.08	0.51	6.04	4.00	-	-	-
High	321	47.05	0.36	10.02*	7.97*	3.98*	-	-
Highest	107	50.62	0.62	13.58*	11.54*	7.54*	3.57*	-

M, mean; SE, standard error

* *p* < 0.05

## Discussion

The purpose of the study was to investigate the web-based PCI’s effectiveness on tourism workers’ mental well-being. The study revealed that after 1 month, the mental well-being of the workers increased at a statistically significant level. The changes strengthened the effectiveness of a web-based PCI. Its success was due to the design, creation, and development process that was rooted in the concept of PsyCap, which consists of hope, self-efficacy, resilience, and optimism. PsyCap was chosen for the intervention as many scholars have studied it and adapted it for use in various settings [19]. Subsequently, PCI was shown to be able to alleviate mental well-being problems such as depression; enhance positive mental health and appropriate work behavior [17]; improve psychological well-being (for example, increased work satisfaction) [21]; and enhance organizational behaviors, like lower turnover intentions [23], increased job engagement [27], and reduced organizational procrastination [28]. For these reasons, the intervention developed from the PsyCap concept could help tourism workers increase their mental well-being.

In addition, the intervention was developed to specifically enable individuals to manage or adapt to the COVID-19 pandemic, a volatile, complex, and ambiguous situation. Luthans and Broad [13] proposed that applying the PsyCap concept and developing a PCI can mitigate mental health problems such as stress, anxiety, depression, and posttraumatic stress disorder, all of which can lead to suicide. According to Alat et al. [29] found that PsyCap alleviate the Indian citizens’ psychological distress during COVID-19 lockdown. The content and activities of our program were designed to be consistent with the structure and elements of PsyCap. Resilience-building is one of the strengths of our program. This activity drew upon the resource realization technique, which we employed in the “My character” survey to help people bounce back from their life crises. Hope was fostered by having individuals set achievable goals in the “My goals” activity. Our qualitative data revealed that entrepreneurs were inspired, developed appropriate behaviors, and persevered when they saw that others who had encountered problems got through or did not give up, with their problems seemingly easing. These reactions reflect the effects of the vicarious learning and social influence that were promoted through the self-efficacy training presented in the “My idol” activities. Furthermore, the intervention used emotion induction techniques by collecting and presenting activities the individuals had done before in a short video clip. Visual stimuli, music listening, and autobiographical recalls were used so that individuals could review their growth. The techniques allowed the individuals to learn, draw inspiration, and develop positive feelings. This approach is consistent with a study by Siedlecka & Denson [30], who suggested that visual stimuli, music listening, and autobiographical recall can induce happiness. Those researchers found that combining those methods with other techniques significantly increased the effectiveness of their training activities. In addition, the intervention focuses on allowing users to explore and review themselves in their current state and become aware of emotions and feelings present at that moment. Thinking, anticipating, and planning based on what is real without judging, overgeneralizing, or forming catastrophic thoughts is consistent with the principles of mindfulness. This approach is supported by investigations of factors that may promote good mental health or alleviate mental suffering during the COVID-19 pandemic [31, 32]. In the field of PsyCap, mindfulness is considered a dominant predictor of negative affects and is already regarded as the fifth component of PsyCap [33].

However, this is one of the first web-based PCIs employed in Thailand. It is an online, individualized, self-learning platform, unlike the original technique that uses a group process and individual counseling through a face-to-face method. This self-learning method creates a sense of autonomy through the self-management of the learning process [34]. A previous study investigating PCI effectiveness through various platforms found that a web-based PCI is more effective than face-to-face methods [22], can increase work satisfaction, and can reduce the likelihood of an individual deciding to resign from a permanent position [23].

Since the web-based PCI was developed from the concept of PsyCap, substantial research supports that it can relieve mental well-being problems and promote positive mental health. Therefore, the web-based PCI can be applied to the COVID-19 situation people face. The intervention was designed as an online self-learning platform based on tourism workers’ data and was validated by several experts. The program proved to be effective in improving mental well-being, particularly when applied frequently. However, there are 2 major limitations to implementing the results of this study and planning for future studies. The first limitation is the lack of a control group. Consequently, the program’s effectiveness cannot be separated from other relevant variables. To address this limitation, there should be a control group that may use the same program but is conducted face-to-face or through any activity that educates people about PsyCap. The second limitation is that there was no long-term follow-up on mental well-being. A 3- or 6-month follow-up might have confirmed the development of mental well-being and the effectiveness of the approach in improving mental well-being.

## Conclusions

This study developed a web-based PCI that focused on developing the attributes of hope, self-efficacy, resilience, and optimism, which are key elements of positive psychological capital (PsyCap). It was found that the web-based PCI can be used to improve the mental well-being of tourism workers. The implications of this research are that a web-based PCI can be an effective tool for improving the mental well-being of tourism workers who have been negatively impacted by the COVID-19 pandemic. However, future studies should include a control group in the research design to further support the credibility of the web-based PCI in improving the mental well-being of tourism workers. This would help ensure that any improvements in mental well-being are due to the intervention and not by other factors. Additionally, future studies could explore the long-term effects of the intervention and whether it can be applied to other industries beyond tourism.

## Data Availability

The data that support the findings of this study are available from the corresponding author upon reasonable request.
